# Book Reviews

**Published:** 1914-09

**Authors:** 


### BOOK REVIEWS

---

Books mentioned may be inspected at and ordered through this office. So far as possible, books received in any month will be reviewed in the issue of the second month following.

---

**Practical Medicine Series,** comprising ten volumes on the year's progress in medicine and surgery, under the general editorial charge of Charles L. Mix, A. M., M. D., and Roger T. Vaughan, Ph. B., M. D.; published by the Year Book Publishers, 327 S. La Salle Street., Chicago. Price for the series of ten volumes, $10.

Vol. 1. General Medicine, edited by Frank Billings, M. S., M. D. and J. H. Salisbury, A. M., M. D. 388 pages, $1.50 separately.

Vol. 2. General Surgery, edited by John B. Murphy, A. M., M. D., LL. D., F. R. C. S., F. A. C. S. 608 pages, $2.00 separately.

Vol. 3. Eye, Ear, Nose and Throat, edited by Casey A. Wood, C. M., M. D., D. C. L., Albert H. Andrews, M. D., and William L. Ballenger, M. D. 366 pages, $1.50 separately.

This series, appears this year in black covers, to distinguish from those of previous years. The size is uniform. Current literature is reviewed in a highly satisfactory way, many of the articles being illustrated, some in colors, and the reviews of new methods and subjects, as electro-cardiography, are very lucid. We confess that, in the past, we have read many articles on such subjects and have failed to grasp the general principles involved until they have been cleared up in this series.

---

**On Dreams,** by Prof. Dr. Sigm. Freud, only authorized English translation by M. D. Eder, from the second German edition, with introduction by W. Leslie Mackenzie, M. A., M. D., LL. D.; published by the Redman Co., Herald Square Bldg., New York City. Cloth, 110 pages, $1.00.

This work has received so much criticism, favorable and unfavorable, that the contents are already quite familiar. To have Dr. Freud's presentation of the subject, at first hand, in an authorized translation, is better than to receive ideas at second hand with the tinge of the various critics.

---

**Ambidexterity and Mental Culture,** H. MacNaughton-Jones, M. D., M. CH., Q. U. I., M. A. O. R. U. I., F. R. C. S. I. and Ed., London; published by the Rebman Co., Herald Square, New York City. 102 pages, 75 cents.

The author assumes that primitive man was ambidexterous and while the preference for the right hand occurs in practically all peoples and for all historic time, its explanation is not complete. In the young infant, the differentiation does not exist. Forcible correction of left-handedness is opposed but the author strongly advises education in ambidexterity and describes the Montessori system. Numerous interesting examples of acquired left handedness after loss of the right hand, and a great many other details, are given. The reviewer may be pardoned for urging the importance of ambidexterity upon the medical profession and, at the same, stating his personal compromise. Originally, normally right handed, as a boy, he made strenuous efforts toward ambidexterity without great success. In learning a new muscular act of any kind, he endeavors to use either hand at will, or rather as theoretically more convenient, for example, he shaves each side of the face with the nearer hand. For writing, cutting and other finely co-ordinated actions, the right hand is used unless disabled. For type-writing and other acts not requiring fine adjustment, both hands are used as nearly equally as possible. To offset the fact that so many mechanic appliances are constructed so as to render the use of the left hand awkward, all coarse muscular acts are performed, so far as possible with the left arm and hand, not so much in the interests of ambidexterity in the true sense as to avoid muscular asymmetry.

---

**Local Anaesthesia,** Dr. Arthur Schlesinger of Berlin, translated by F. S. Arnold, B. A., M. B., B. Ch., (Oxon.); published by the Rebman Co., Herald Square Bldg., New York City. 211 pages, $1.50.

The history and physical principles involved are first discussed. The agents used are cocaine and its substitutes and cold, cold as well as anaemia being also considered as adjuvants to pharmacal anaesthetics. Anaemia may be produced by constriction, infiltration (Schleich method), and suprarenal preparations. Nerve blocking, venous and arterial anaesthesia are also considered. The technique is discussed in detail, for various regions, including the extention of local anaesthesia to abdominal operations, etc. The antiquity of some of the means employed to secure local anaesthesia, is both interesting and surprising.

---

**A Message of Health,** Russell C. Markham, M. D., Marquette, Mich., published by Boericke & Tafel, Philadelphia, 123 pages, 75 cents.

This book consists of a series of essays, on blood making, food values, balanced meals, a vegetarian pugilist, the law of similars, from Moneron to man in God's image, the conquest of fear or worry, disarming the doubter and various others. It is not a scientific work nor systematic but it does not profess to be either. There are plenty of technical books and plenty of lengthy treatises in which medical technicalities are reduced to the "comprehension of the meanest reader" as one quaint old theologic work puts it. There are few books of this sort in which the author, briefly and without attempt to duplicate scientific knowledge easily accessible, touches on many phases of life in a suggestive and helpful way, without interfering with the reader's own views, or without directly stimulating him to antagonistic views. We have nearly lost the art of helpful conversation, doing our hard thinking and studying in private. This book is really one side of that sort of conversation which the ancients used so largely as an intellectual help.

---

**Practical Hormone Therapy,** a Manual of Organo-therapy for General Practitioners, by Henry R. Harrower, M. D., New York, with fore-word by Prof. F. Dr. Artur Biedl of Vienna, published by Paul B. Hoeber, 69 E. 59, N. Y., 488 pages.

This is the first comprehensive treatise on the subject, from the practical medical standpoint, irrespective of commercial catalogues, that has been published in any language, so far as we know. The author studied the subject in a number of laboratories, personally visited the prominent investigators of the general subject and secured their criticisms and revision. By this method he secured personal familiarity with technical details, acquired a judicial attitude and, at the same time, modestly sacrificed a claim for originality to the higher end of securing the broadest and most authoritative information on the subject. Biedl, Beebe, Bayle, Berkeley, Carnot, Ewald,

Hertoghe, Kelly, Lepine, Murray, Sajous, Schaefer, Starling, Williams and Zuelzer have thus set their seal on practically all the chapters dealing with technical details and controversial points. For the chapter on secretins and pyloro-duodenal extracts, he secured the criticism of three of these experts. Extensive bibliographies are given at the end of many chapters, as well as a list of text books in various languages and of manufacturers' literature, for the general subject. Ten pages are devoted to a glossary of terms beside a general index of 13 pages of fine print.

Hormones are classified as of the digestive system, the metabolic glands, the nervous system, the reproductive system, the vascular system, and miscellaneous, including the use of pluriglandular extracts, the control of hyperendocrinism, the lipoids, etc. The number of individual hormones and the variety of clinical conditions in which they have a practical part, will surprise those who have not followed this subject closely. At the same time, the author has divested himself of a partisanship which would lead to making sweeping claims and result in disappointment.

We feel justified in asserting that this book marks an epoch in medical history. On the one hand, it places the hormones in the hands of the profession as part of its therapeutic armamentarium whereas, up to this time, they have been limited mainly to interesting items of the physiologist. On the other hand, it both elevates them from the commercial to the scientific level and adds immensely to their commercial importance. The work is timely in no trite sense. The author has waited till there has accumulated a considerable amount of scientific information, sufficient to justify practical utilization of the theoretic principles and observations. He has brought matters to the bar of professional trial with a full presentation of existing evidence, pro and contra. It is too early to prophesy just how important a place hormone therapy will assume. It is too much to expect that the entire fabric of scientific observation and theory will prove durable. It is only too obvious that it is imperfect in many particulars and the author deserves great credit for not having let enthusiasm lead him to supply deficiencies with hypotheses derived solely from speculation. But this work systematizes and collates all the fundamental details known, and extends the bounds of practical therapeutics to a considerable degree, and affords a basis for future clinical investigation. It is a book for which an immediate demand exists and one for which we anticipate a progressive existence through new editions as new facts and experience are accumulated.

**Diseases of the Rectum and Colon and Their Surgical Treatment,** Jerome M. Lynch, M. D., New York City, Lea & Febiger, Philadelphia and N. Y., 596 pages, 228 engravings and 9 colored plates, $5.00.

This is a complete systematic treatise of the highest standards, literary, scientific, and as representative of the publisher's and printer's art. One of the plates reproduces in color, endoscopic appearances and there are several radiographs. The practical conduct of examinations, by endoscopes, ordinary specula, palpation, etc.; the preparation and after care of patients, and other details of equal importance are discussed under special headings. We note with pleasure the directions as to carefulness in the passage of endoscopes though we prefer to pass such instruments the full distance with the obturator so as to allow inflation or injection of purpetrol as neccessary to assist in the passage, without removing the obturator. We also prefer an independent, removable staff bearing the electric light, instead of the more elaborate device of a special lateral passage for a permanently placed light. To avoid fatigue on the part of the patient, we usually pass the sigmoidoscope in the lateral position. The author ignores the possibility of this method, as do most operators. These expressions of personal, minority views, do not detract at all from our high regard for the work which is most heartily commended to our readers.

---

**Report of U. S. Commissioner of Education,** for the year ended June 30, 1913, Vol. 2.

From this very interesting report we cull the following statistics: There are over 11,000 public high schools and over 2000 private high schools in the U. S. The former have increased from 68% to 88% of the teaching capacity for secondary education since 1890. Of the approximately 1,900,000 children of the age of 14, which may be taken as the average for the first year of the high school course, 513,000 were actually enrolled in high schools. In other words, between a quarter and a third of the rising generation is receiving at least the beginning of a high school education. Over 177,000 completed the high school course—nearly 10% of the population of the age of 18 .

There are about 170,000 persons of the average age of college graduates. 25,824 persons received bachelor's degrees—almost exactly 1.5%. The two sexes are about equally divided in the general population and over 16,000 men received bachelor's degrees, so that we may estimate that, for the whole rising generation, nearly 2% of men and nearly 1.25% of women, have a college education.

While the higher education of the public is commendable, it must not be forgotten that it represents an enormous tax, both in money and in withdrawal of young persons from actual labor. An education that is not used is a waste. Moreover, if more persons are educated to a certain degree of efficiency along clerical and professional lines than are needed, a considerable economic difficulty presents itself. A humorist wrote at the time of the Spanish War of a patriot who offered his sword to his country and had it declined on the ground that the country wanted muskets. It is worth while to inquire seriously whether the possession of an education unfits its possessor for or disinclines him to engage in humble occupations for which such education is not necessary and whether a country can exist without serious economic friction with 2% of its men educated for the higher intellectual pursuits and with 10% of both men and women educated beyond the needs of ordinary labor, and business.

---

**The Digestive Disturbances of Infants.** Schelble, in Deutsche Medizinische Wochenschrift, May 28, 1914, gives an excellent survey of these troubles based upon the present school of pediatrics in Germany, the classification of the disorders according to their etiology makes the subject easy to handle and does away with a good many wrong ideas still prevailing in this country. Amongst other things it proves the slight role played by the bacterial contents of the milk and of the gastro-intestinal tract of the patient.—C. G. L.-W.

---

**Demonstration on Living Subject of Treponema in the Brain of General Paralytics.** Beriel and Durand, Lyon Med., November 23, 1913, refer to the demonstration in the brain at necropsy of treponemata by Noguchi, Moore, Marinesco, A. Marie, Levadati and Bankowski. They puncture the anterior portion of the brain through the sphenoid fossa, withdraw several c. c. of cephalorhachidian fluid, then the needle is advanced quickly several millimeters and suction made by the syringe. In this way, a minute portion of cortex is removed. They have performed the operation five times, in four patients. Twice, in three cases, (sic) very mobile treponemata have been demonstrated by the India ink method. No harm resulted in any case. (Note: Without admitting the presence of treponemata in our own tissues, we do admit a reluctance to losing even a small portion of the brain and we are inclined to agree in advance with what the antis will say about human vivisection.—Ed.)

